# The loss of healthy life time is similarly high for both food allergy and intolerance sufferers

**DOI:** 10.1186/2045-7022-5-S3-P8

**Published:** 2015-03-30

**Authors:** Andreas Arens-Volland, Norbert Rösch, Sabine Schnadt

**Affiliations:** 1 CRP Henri Tudor, Luxembourg, Luxembourg; 2 University of Applied Sciences, Kaiserslautern, Germany; 3 German Allergy and Asthma Association, Mönchengladbach, Germany

## Background

Little is known about the loss of productive workforce or healthy life time due to food allergy or food intolerance. One aim of the BELANA trial (Burdens and Expenses of Living as Adult with Nutrition based Allergy or Intolerance) was to investigate the healthy time lost for work or spare time due to corresponding disease.

## Methods

314 study participants (≥18) with self-reported food allergies or intolerances were recruited in 2009 by the German Allergy and Asthma Association (DAAB). 247 completed the BELANA questionnaire four times within four-month intervals. The participants had to state whether they were unable to fulfil their daily tasks at work, at home, or at school due to problems with their food allergy or intolerance. Furthermore, they had to indicate the number of affected days.

## Results

**Figure 1:**
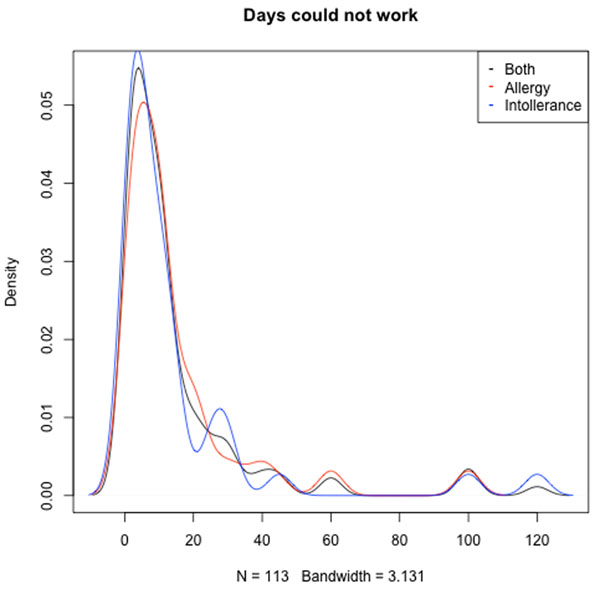


About 43% (average of all single surveys) of all self-reported food allergy and food intolerance suffers reported that they have missed at least one day of work or every day life during the past four months. About 37% of the participants stated to not have experienced such losses. Comparing the two subgroups of food allergic and food intolerant persons, no significant difference could be determined. Similarly, reviewing the actual number of days lost, people from both subgroups accounted for a noticeably high number of lost days: 15.68 days on average during the past four months. Figure 1 exemplarily shows the density distribution of lost workdays for the participants of the fourth iteration of the questionnaire.

## Conclusion

The group of self-reported food allergy and food intolerance sufferers that complain about the loss of healthy time due to their illness is larger as compared to the group that does not experience this kind of burden. The high number of lost healthy days indicates that the data might be biased towards severe cases of food allergy and intolerance. This will need further investigation through cross-validation with other questionnaire items.

